# Deviation of the typical AAA substrate-threading pore prevents fatal protein degradation in yeast Cdc48

**DOI:** 10.1038/s41598-017-05806-y

**Published:** 2017-07-14

**Authors:** Masatoshi Esaki, Md. Tanvir Islam, Naoki Tani, Teru Ogura

**Affiliations:** 1 Department of Molecular Cell Biology, Institute of Molecular Embryology and Genetics, Kumamoto University, Kumamoto, 860-0811 Japan; 2 Core Research for Evolutional Science and Technology, Japan Science and Technology Agency, Saitama, 332-0012 Japan; 3 Program for Leading Graduate Schools “HIGO Program”, Kumamoto University, Kumamoto, 860-8556 Japan; 4 Department of Microbiology, Jessore University of Science and Technology, Jessore, 7408 Bangladesh; 5 Liaison Laboratory Research Promotion Center, Institute of Molecular Embryology and Genetics, Kumamoto University, Kumamoto, 860-0811 Japan

## Abstract

Yeast Cdc48 is a well-conserved, essential chaperone of ATPases associated with diverse cellular activity (AAA) proteins, which recognizes substrate proteins and modulates their conformations to carry out many cellular processes. However, the fundamental mechanisms underlying the diverse pivotal roles of Cdc48 remain unknown. Almost all AAA proteins form a ring-shaped structure with a conserved aromatic amino acid residue that is essential for proper function. The threading mechanism hypothesis suggests that this residue guides the intrusion of substrate proteins into a narrow pore of the AAA ring, thereby becoming unfolded. By contrast, the aromatic residue in one of the two AAA rings of Cdc48 has been eliminated through evolution. Here, we show that artificial retrieval of this aromatic residue in Cdc48 is lethal, and essential features to support the threading mechanism are required to exhibit the lethal phenotype. In particular, genetic and biochemical analyses of the Cdc48 lethal mutant strongly suggested that when in complex with the 20S proteasome, essential proteins are abnormally forced to thread through the Cdc48 pore to become degraded, which was not detected in wild-type Cdc48. Thus, the widely applicable threading model is less effective for wild-type Cdc48; rather, Cdc48 might function predominantly through an as-yet-undetermined mechanism.

## Introduction

ATPases associated with diverse cellular activities (AAA+) proteins convert the chemical energy of ATP to mechanical forces to carry out diverse functions such as unfolding of their substrate proteins, disassembly of protein complexes and microfibers such as microtubules and amyloid fibrils, and the transport of macromolecules by walking on cytoskeletons, among others^1–3^. AAA proteins are defined as a subfamily of AAA+ proteins based on possession of the second region of homology (SRH) domain^4^. Bacteria and archaea harbor a few AAA proteins, most of which function as unfoldases. Eukaryotes contain 20–24 highly conserved AAA proteins, which function at multiple intracellular regions, including the nucleus, mitochondria, and cytosol. All AAA+ proteins contain one or two AAA+ modules as a mechanochemical engine, which typically form a hexameric ring structure with a central narrow pore. The ring structure has been shown to be essential for ATP hydrolysis and the proper functions of many AAA+ proteins.

In order to uncover the nature of biological reactions, the underlying fundamental mechanisms as well as the molecular architectures of essential enzymes must be elucidated. A threading mechanism has been proposed as a key and common molecular mechanism driving the reactions of several AAA+ proteins, which was mainly based on data obtained using bacterial AAA+ module-regulated proteases^5^. The bacterial AAA protease FtsH forms a AAA hexameric ring followed by a proteolytic chamber^6^. Since the AAA pore is interconnected to the interior of the proteolytic chamber, substrate proteins must first pass through the narrow pore of the AAA ring to reach the proteolysis site to be degraded. During such threading through the AAA pore, substrate proteins undergo unfolding by a function of the AAA module. The loop structure protruding into the pore (pore loop) is essential for this threading mechanism, especially the highly conserved motif sequence ΦXG (an aromatic amino acid, any residue, and a glycine residue; see Supplementary Fig. S1), since replacements of the first and third residues of the ΦXG pore loop in FtsH with alanine drastically eliminate its unfolding activities^7^. The aromatic residue in the ΦXG motif pore loops of two AAA+ modules in ClpB has been shown to interact directly with substrate proteins^8^. In addition to its function in AAA+ unfoldases, the ΦXG motif is also essential for other types of AAA+ proteins such as microtubule-severing AAA enzymes^9, 10^.

The budding yeast *Saccharomyces cerevisiae* possesses 22 AAA proteins with 25 AAA modules, most of which contain conserved ΦXG motif sequences in their pore loops (Supplementary Fig. S1). An exception is one of the AAA modules of Cdc48. Cdc48—also generally known as p97, VCP in humans, and TER94 in *Drosophila*—is one of the most abundant intracellular proteins. Cdc48 is essential for multiple cellular and molecular events, including mitochondrial protein degradation, endoplasmic reticulum-associated protein degradation, autophagosome formation, homotypic fusion of the endoplasmic reticulum and Golgi membranes, nuclear envelope reassembly, transcription activation, DNA replication, meiotic progression, and modification of protein aggregates^11–13^. In contrast to the breadth of research conducted for unraveling the versatile cellular functions of Cdc48, the underlying molecular mechanisms to explain how Cdc48 mediates these individual reactions remain largely unknown.

**Figure 1:**
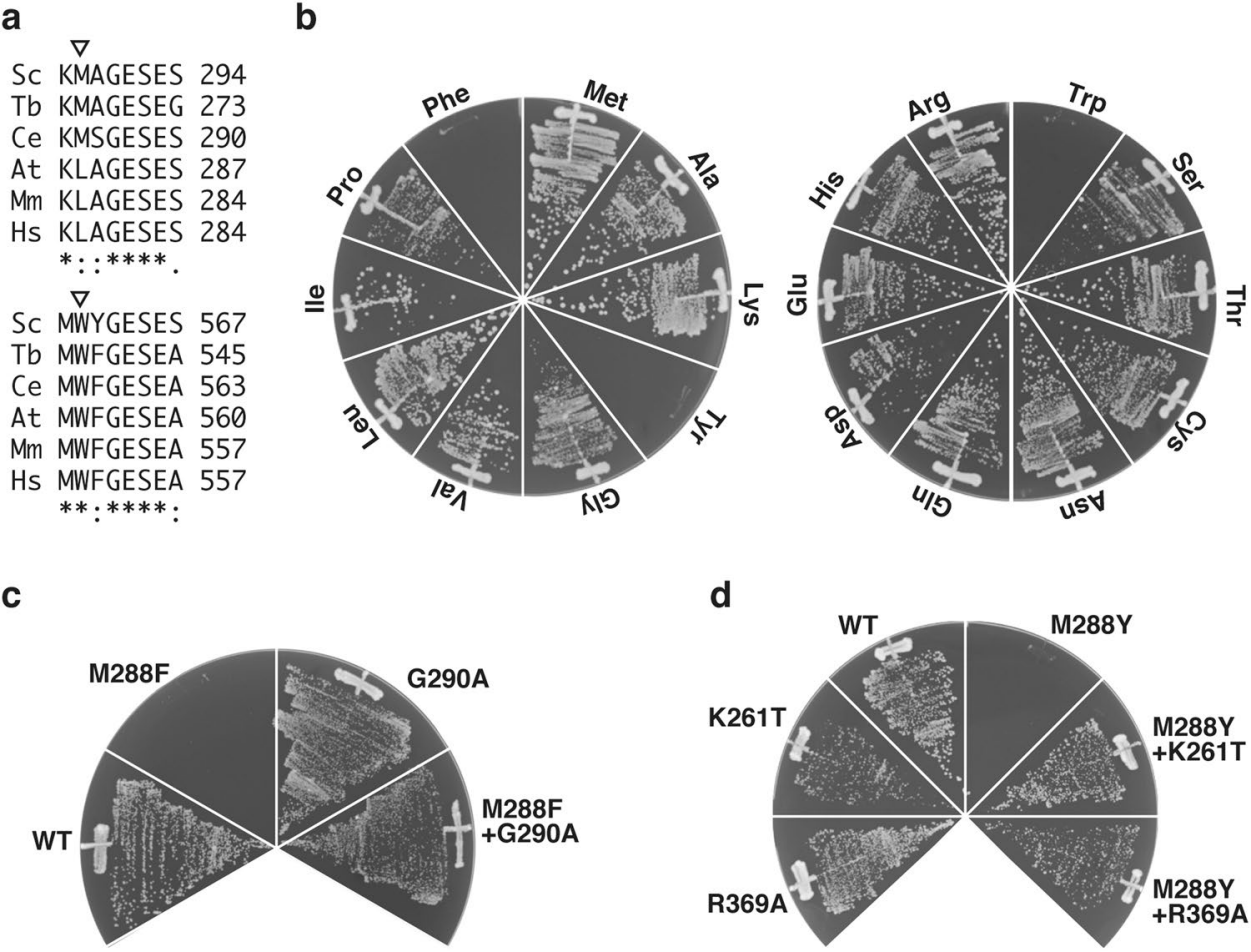
Replacement of the residue 288 of yeast Cdc48 with aromatic amino acids was lethal. (**a**) Eukaryotic Cdc48 homologs were aligned. A whole sequence alignment is shown as Supplementary Fig. S2. The triangles indicate the first positions of the pore loop ΦXG motif sequences of the D1 (upper panel) and the D2 (lower panel) AAA domains. Sc, *Saccharomyces cerevisiae*; Tb, *Trypanosoma brucei*; Ce, *Caenorhabditis elegans*; At, *Arabidopsis thaliana*; Mm, *Mus musculus*; Hs, *Homo sapiens*. (**b**) *cdc48*∆ cells carrying pME351, a *URA3*-based plasmid expressing Cdc48, were transformed with a *HIS3*-based plasmid for the expression of Cdc48 with the indicated amino acid residue at the residue 288. The transformants were grown on media lacking histidine supplemented with 5′-FOA at 30 °C to counterselect against pME351. (**c**,**d**) Tested were viabilities of cells expressing Cdc48 with the indicated mutations as shown above.

Cdc48 is composed of an N-terminal regulatory domain followed by two AAA domains^14, 15^. The C-terminal AAA domain (D2) of Cdc48 executes the major ATPase activity and is essential for cell growth, whereas the ATPase activity of the N-terminal AAA domain (D1) is dispensable for cell growth but modulates the activity of the D2 domain^16, 17^. The ΦXG motif sequence is perfectly conserved in the D2 pore loop in all eukaryotic homologs of Cdc48, whereas the corresponding first position of the D1 pore loop is exclusively occupied by a non-aromatic amino acid residue (Fig. 1a, Supplementary Fig. S2). The second residue of the D1 pore loop of Cdc48 is a small amino acid, whereas bulky or aromatic amino acid residues occupy the second position of the ΦXG motif of typical AAA proteins. Archaebacteria such as *Thermoplasma acidophilum* and *Methanococcus jannaschii* have a close homolog of Cdc48, including the ΦXG motif sequence in the D1 pore loop. Therefore, it is likely that the ΦXG motif sequence was eliminated from the eukaryotic Cdc48 homologs through evolution. Interestingly, replacement of the non-aromatic leucine residue in the D1 pore loop of mouse p97 with tyrosine endowed the enzyme with extrinsic unfolding activity *in vitro*, although this only occurred when the N-terminal regulatory domain of p97 was concomitantly removed^18, 19^. This *in vitro* extrinsic unfolding activity seems to be achieved by threading the substrate proteins through the D1 pore. However, the biological significance of these findings and the actual functional mechanism of Cdc48 remain to be clarified because of the lack of supporting *in vivo* analyses.

In the present study, we attempted to clarify the biological significance of the conserved elimination of an aromatic amino acid residue from the D1 pore loop of yeast Cdc48. First, we established Cdc48 mutants with the ΦXG motif sequence in the D1 pore loop, which caused a lethal phenotype. To explore the underlying mechanism and molecules contributing to the effects of the ΦXG motif mutation, we also constructed yeast strains with disruption of a non-essential Cdc48 cofactor gene and those with a known defective mutation in an essential cofactor gene. Moreover, we examined the potential role of the interaction of Cdc48 with the proteasome in mediating the lethality of the mutation.

The proteasome is the major machine for robust protein degradation in the cytosol and the nucleus. The eukaryotic proteasome is mostly present as the 26S complex, which is composed of 19S regulatory particles and a 20S core peptidase (20S proteasome)^20^. The 19S particles contain substrate-recognition and -processing subunits, as well as a AAA hexameric complex, which facilitates unfolding of the substrate. The 20S proteasome forms a barrel-like structure with proteolysis-active sites inside the barrel. Substrate proteins reach the inside of the proteolytic chamber through narrow axial channels. The gates to the chamber are strictly regulated and are closed to avoid abnormal protein degradation. One of the keys to open the gates of the proteolytic chamber is an HbYX (a hydrophobic amino acid, tyrosine, and any residue) motif at the C-termini of AAA proteins of the 19S particles. The AAA ring of the 19S regulatory particles docks onto the 20S gate, unfolds substrate proteins by threading through the AAA ring, and sends the substrates into the proteolytic chamber of the 20S proteasome. In general, Cdc48 functions upstream of the 19S regulatory particles of the proteasome and delivers substrate proteins to the 19S particles^21^. The sequence of the C-termini of all eukaryotic Cdc48 homologs, LYX, is consistent with the key for the HbYX gate to open the proteolytic chamber (Supplementary Fig. S2), and a Cdc48 homolog was recently shown to functionally unlock the gate of the 20S proteasome without the 19S particles *in vitro* ^19, 22^. Therefore, we hypothesized that the lethality of the Cdc48 mutant may be related to abnormal regulation of the unfolding of certain proteins, so that the unfolded proteins are sent directly to the 20S proteasomal chamber to be degraded. We tested this hypothesis by exploring the possibility of a direct interaction between the Cdc48 mutant and the 20S proteasome, and the consequence of this interaction on the lethal phenotype caused by the mutations.

Finally, we conducted a proteomics analysis to identify any candidate substrates of the Cdc48 mutant and determine its molecular effects on protein degradation and stability. Together, these experiments should help to clarify the function of the unique pore loop in Cdc48.

## Results

### Introduction of an aromatic amino acid into the D1 pore loop of yeast Cdc48 is lethal

To explore the reason for the apparent evolutionary elimination of the aromatic amino acid residue from the first position of eukaryotic Cdc48, we constructed yeast strains with disruption of the genomic *CDC48* gene expressing a mutant Cdc48 under the control of the authentic promoter from a single-copy plasmid^16^. Similar to the lethal ATPase activity-deficient mutations K534A and E588Q in the D2 domain^16^, replacement of Trp561 at the first position of the ΦXG motif of the D2 pore loop with an alanine residue was found to be lethal (Supplementary Fig. S3a). Interestingly, replacements of Met288, which corresponds to the first residue of the D1 pore loop, with phenylalanine, tryptophan, and tyrosine residues, but not the other 16 amino acid residues, were also lethal (Fig. 1b). Gly290 of the third residue of the D1 pore loop could be replaced with alanine without any obvious growth defect (Fig. 1c). By contrast, the replacement of Gly290 with alanine suppressed the lethal defect due to the M288Φ mutations (Fig. 1c). Furthermore, introduction of ATPase-deficient mutations such as K261T and R369A^16^ in the D1 domain also suppressed the lethality of the M288Φ mutations (Fig. 1d). An E588D mutation, which severely, but not completely, inhibited the ATPase activity of the D2 domain, failed to suppress the lethality of the M288Φ mutations (Supplementary Fig. S3b). These results suggest that the features required for the threading mechanism to operate, i.e., the ΦXG pore loop and ATPase activity, in the D1 domain of yeast Cdc48 are toxic for vegetative growth. This finding suggests a possibility that similar to other AAA modules, the ATPase-dependent movements of the mutated D1 may facilitate the unfolding of some proteins by pulling them into the pore, thereby perturbing essential cellular processes.

### Ufd2 and Shp1 are predominantly responsible for the lethal phenotype

**Figure 2:**
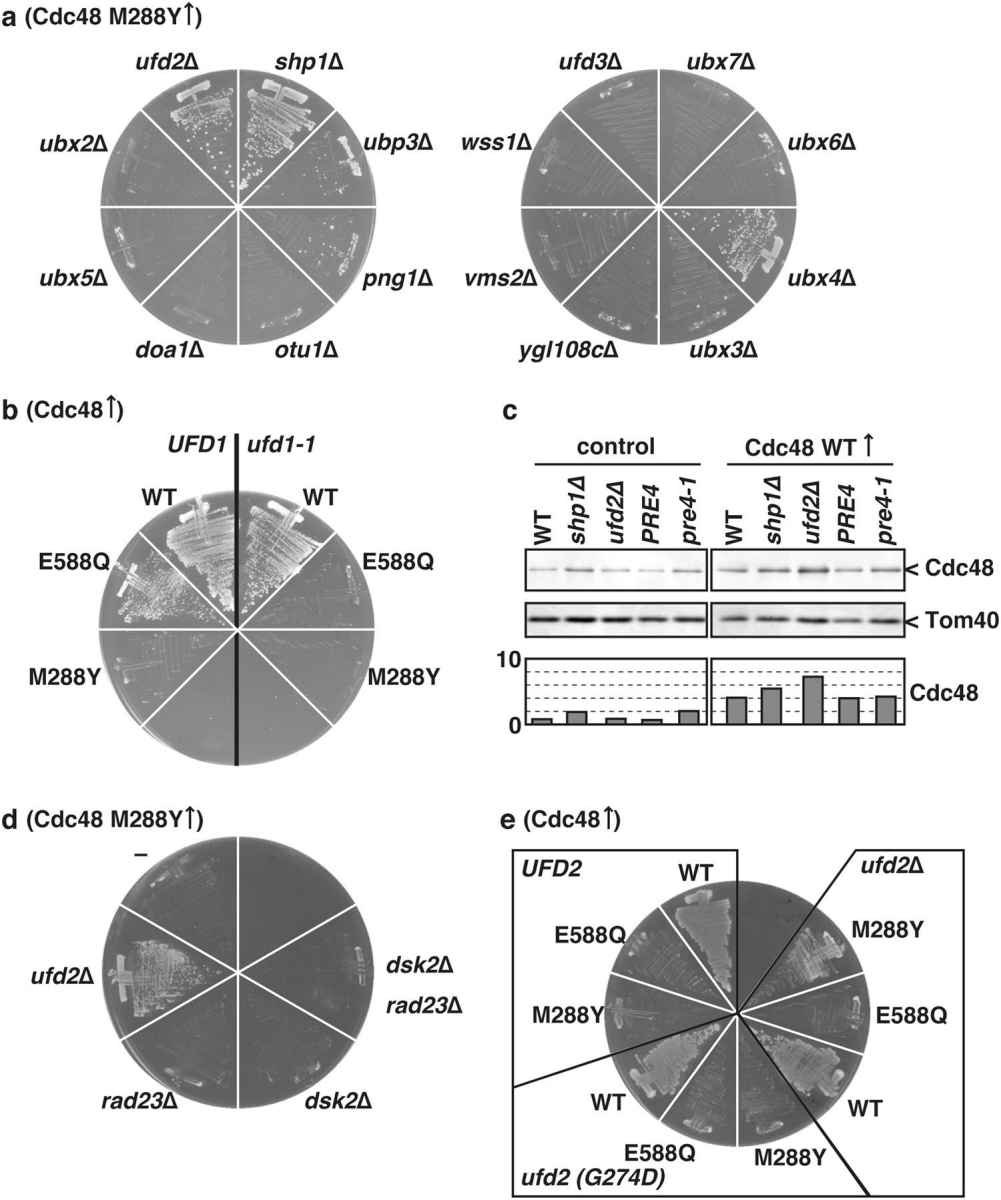
*UFD2* and *SHP1* were predominantly involved in expressing lethal phenotype by the M288Y mutation of Cdc48. (**a**) Cells lacking the indicated endogenous gene were transformed with a plasmid expressing the Cdc48 M288Y mutant under the control of the *CUP1* promoter. The transformants were grown on media supplemented with 10 µM CuCl_2_ at 30 °C for 2 days. (**b**) *ufd1-1* cells and the corresponding wild-type *UFD1* cells were transformed with a *CUP1* promoter-regulated plasmid expressing wild-type Cdc48 (WT) and mutant Cdc48 with the replacement of E588Q and M288Y. The transformants were grown on media supplemented with 10 µM CuCl_2_ at 30 °C for 2 days. (**c**) Whole cell lysates were prepared from cells with a plasmid expressing wild-type Cdc48 (right panel) and no proteins (left panel) grown at 10 µM CuCl_2_ as shown previously^48^. Equal amounts of cell extracts were analyzed by SDS-PAGE followed by immunoblotting using anti-Cdc48 and anti-Tom40 antibodies. The graphs show relative amounts of Cdc48 using Tom40 as a control. (**d**) Cells lacking the indicated endogenous genes were transformed with the *CUP1* promoter-regulated plasmid expressing the Cdc48 M288Y mutant. The transformants were grown on media supplemented with 100 µM CuCl_2_ at 30 °C for 2 days. (**e**) *ufd2*∆ cells were transformed with a plasmid expressing wild-type Ufd2, Ufd2 with a G274D mutation, no proteins. Those cells were transformed with the *CUP1* promoter-regulated plasmid expressing wild-type Cdc48 (WT) and mutant Cdc48 with the replacement of E588Q and M288Y. The transformants were grown on media supplemented with 100 µM CuCl_2_ at 30 °C for 2 days.

Cdc48 plays essential roles in multiple cellular processes, most of which require cofactor proteins for substrate recognition and processing^12, 23, 24^. These cofactor proteins directly bind to their substrate proteins as well as the N-terminal domain or the C-terminal region of Cdc48 through Cdc48-interacting motifs. To explore the potential involvement of Cdc48 cofactor proteins in the M288Φ lethal phenotype, we constructed yeast strains with disruption of a non-essential Cdc48 cofactor gene and those with a known defective mutation in an essential cofactor gene. However, none of the strains with the Cdc48 M288Y mutation could survive (data not shown). Therefore, we constructed another yeast system that allows for regulated expression of Cdc48 mutants in addition to the authentic wild-type Cdc48. We constructed single-copy plasmids expressing a Cdc48 mutant protein under the control of the copper-inducible *CUP1* promoter. When we introduced the plasmids into the wild-type yeast strain, severe growth defects were observed upon induction of the expression of the Cdc48 M288Y mutant in the presence of Cu^2+^ (Supplementary Fig. S4). After transforming Cdc48 cofactor-deficient strains with the plasmid expressing the M288Y mutant, we observed that cells without *SHP1*, *PNG1*, *UBP3*, *UBX4*, or *UFD2* could grow in the presence of the copper-induced M288Y mutant (Fig. 2a,b). Among these strains, those with deletion of *UFD2* or *SHP1* showed the most efficient suppression of the M288Y-induced growth defect (Supplementary Fig. S5), although the induction efficiency of Cdc48 was not affected (Fig. 2c).

Shp1 interacts with the N-terminal domain of Cdc48 and acts as a substrate for recruiting cofactors in protein quality control pathways; however, its detailed functions have not yet been established^25–27^. Ufd2 directly interacts with the C-terminal portion of Cdc48 and functions as an E4 ubiquitin ligase, which elongates the ubiquitin chains attached to the substrate proteins. Dsk2 and Rad23 function downstream of Ufd2 and ferry the polyubiquitinated substrate proteins to the 26S proteasome to be degraded^28, 29^. Surprisingly, deletion of *rad23* and *dsk2* showed no positive effect on suppression of the M288Y growth defect (Fig. 2d). Not only wild-type Ufd2 but also the Ufd2 G274D mutant, which is defective in *in vitro* interaction with Cdc48^30^, was functional in the context of the M288Y growth defect (Fig. 2e). Taken together, these results suggest that Ufd2 is mainly responsible for the growth defect caused by the Cdc48 M288Φ mutant, but likely through a different mechanism than that of the conventional 26S proteasomal protein degradation pathway.

### Features required for complex formation of Cdc48 with the 20S proteasome are responsible for the lethal phenotype

**Figure 3:**
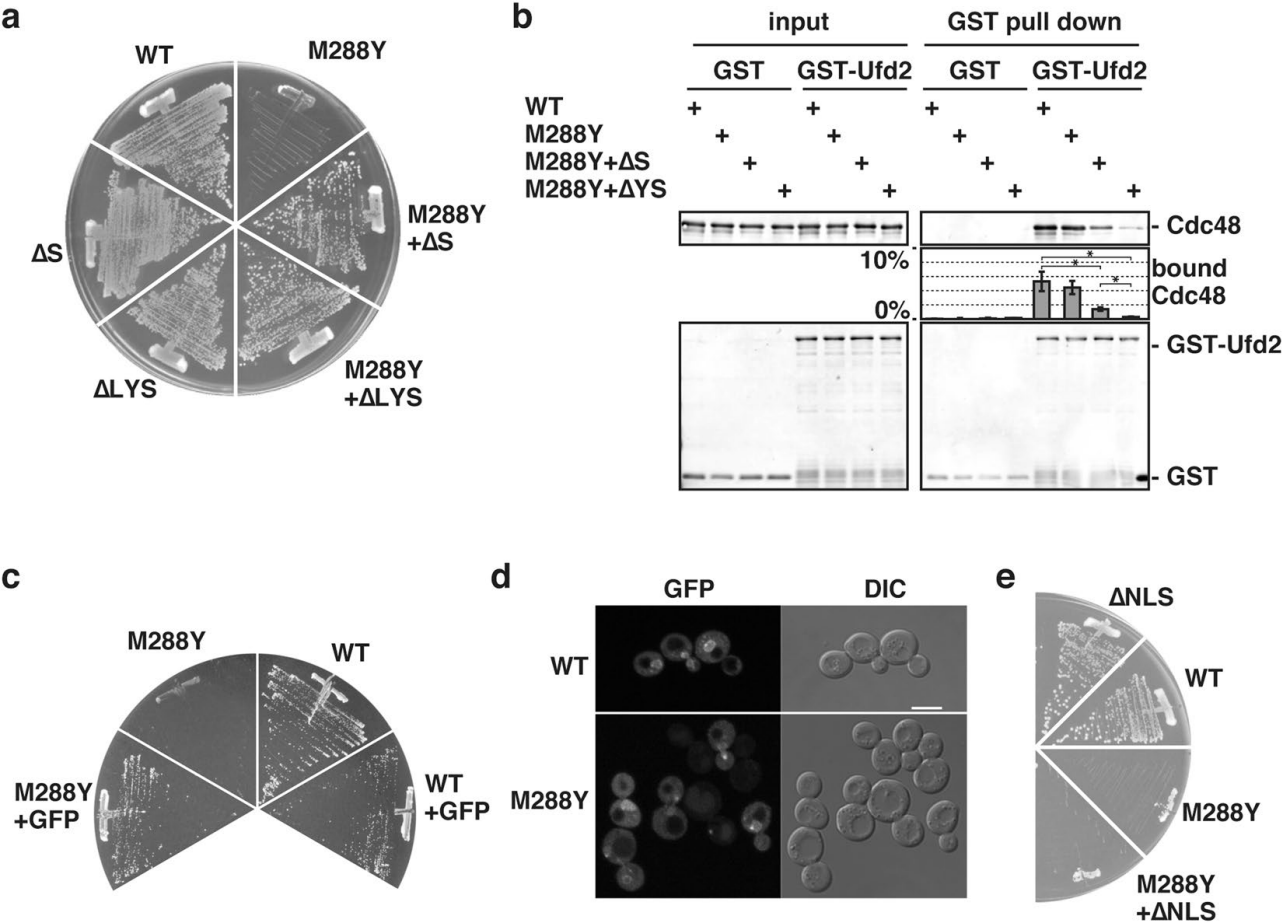
Mutations inhibiting interaction with the 20S proteasome rescued the M288Φ lethal phenotype. (**a**) Tested were viabilities of cells expressing Cdc48 with the indicated mutations as shown in Fig. 1b. ∆S and ∆LYS, deletion of the C-terminal 1 and 3 amino acid residues of Cdc48, respectively. (**b**) GST and GST-Ufd2 were incubated with Cdc48 for 10 min. The GST proteins were isolated using glutathione beads, and eluted proteins from the beads were analyzed by SDS-PAGE followed by immunoblotting using anti-Cdc48 and anti-GST antibodies. Amounts of bound Cdc48 were quantified. input, 5% of GST pull down. ∆YS, deletion of the C-terminal 2 amino acids residues. Averages and standard errors from three independent experiments were shown. Asterisks indicate significant differences with P < 0.05 by student t-test. (**c**) Tested were viabilities of cells expressing untagged Cdc48 and C-terminally GFP-attached Cdc48 with and without the M288Y mutation as shown in Fig. 1b. (**d**) Whole GFP signals within cells were collected by Z-scan by confocal fluorescence microscopy and projected into a 2D image. Representative GFP signals with bright field images (DIC) were shown. bars, 5 µm. (**e**) Tested were viabilities of cells expressing Cdc48 with the M288Y mutation and a loss-of-function mutation in the nuclear localization signal (∆NLS) as shown in Fig. 1b.

Deletion of the last residue, i.e., “X” in the HbYX motif, as well as lack of the complete HbYX motif sequence at the C-terminus of Cdc48 completely suppressed the M288Φ lethal phenotype (Fig. 3a, Supplementary Fig. 6a). These observations suggested that a direct interaction with the 20S proteasome may be required for the lethal phenotype caused by the M288Φ mutations.

Since the Tyr834 residue in the motif of Cdc48 was previously shown to be involved in the interaction with Ufd2^31^, we evaluated the interaction between Ufd2 and Cdc48 without the last Ser835 residue. Wild-type Cdc48 as well as the Cdc48 M288Y mutant efficiently interacted with Ufd2 *in vitro* (Fig. 3b). Deletion of both Tyr834 and Ser835 (∆YS) almost completely abolished the interaction with Ufd2^31^, whereas the single Ser835 deletion mutant retained significant interaction efficiency (Fig. 3b). Neither deletion of the HbYX motif sequence of Cdc48 nor the M288Y mutation decreased the ATPase activity of Cdc48 (Supplementary Fig. S6b). The M288Y mutation also did not change the C-terminal residue-dependent stimulation of the *in vitro* peptidase activity of the 20S proteasome (Supplementary Fig. S6c). Attachment of green fluorescent protein (GFP) for steric hindrance at the C-terminus of Cdc48 also suppressed the M288Φ lethal phenotype (Fig. 3c). Both the wild-type and mutant Cdc48 were predominantly localized in the nucleus and in the cytosol (Fig. 3d). Replacement of the RRKKK sequence beginning at residue 28, which functions as a nuclear localization signal of Cdc48^32^, with RQKNS did not suppress the M288Φ lethal phenotype, suggesting that the lethality largely depends on cytosolic Cdc48 (Fig. 3e).

**Figure 4:**
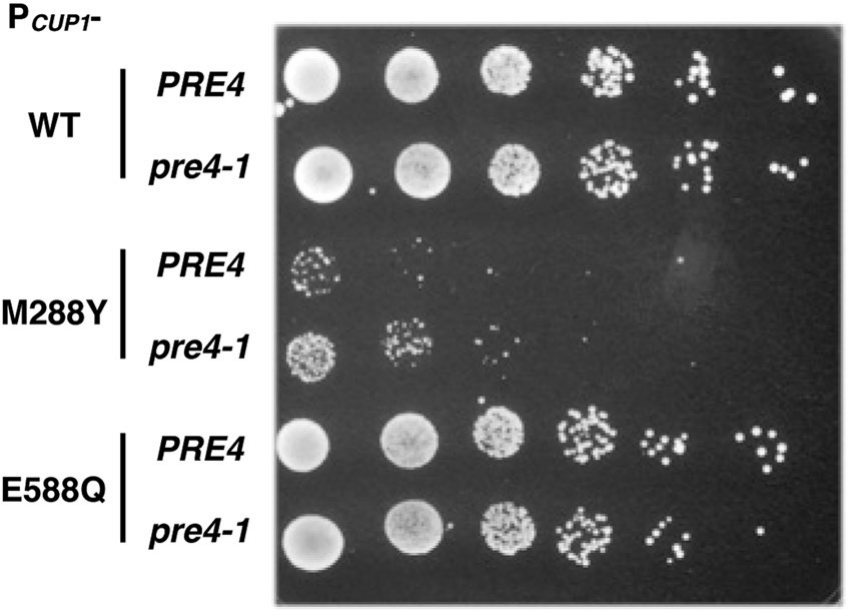
A protease-deficient mutant of the 20S proteasome partially suppressed the M288Φ lethal phenotype. *PRE4* and *pre4-1* cells were transformed with the *CUP1* promoter-regulated plasmid expressing wild-type Cdc48 (WT) and mutant Cdc48 with the replacement of E588Q and M288Y. The transformants were grown on media supplemented with 20 µM CuCl_2_ at 30 °C for 2 days.

To further evaluate the potential involvement of the 20S proteasome on the M288Φ lethal effect, we introduced a mutation (*pre4-1*) into the *PRE4* gene, which encodes the non-catalytic β7 subunit of the 20S proteasome^33^. The *pre4-1* proteasome exhibits a severe defect in one of the three types of protease activity, peptidylglutamyl-hydrolyzing activity^33^. We next transformed the wild-type and *pre4-1* yeast strains with plasmids expressing wild-type and mutant Cdc48 under the control of the *CUP1* promoter. In the presence of 20 µM Cu^2+^, the *pre4-1* mutation relieved the dominant-negative growth defect due to expression of the Cdc48 M288Y mutant (Fig. 4) without affecting the overall expression level of Cdc48 (Fig. 2c). Taking these results together, it is reasonable to assume that the M288Φ lethal phenotype requires an interaction with the 20S proteasome, although the possibility of involvement of the 26S proteasome cannot be completely ruled out at this point.

### Intracellular proteins are degraded by the Cdc48 M288Φ mutant *in vivo*

**Figure 5:**
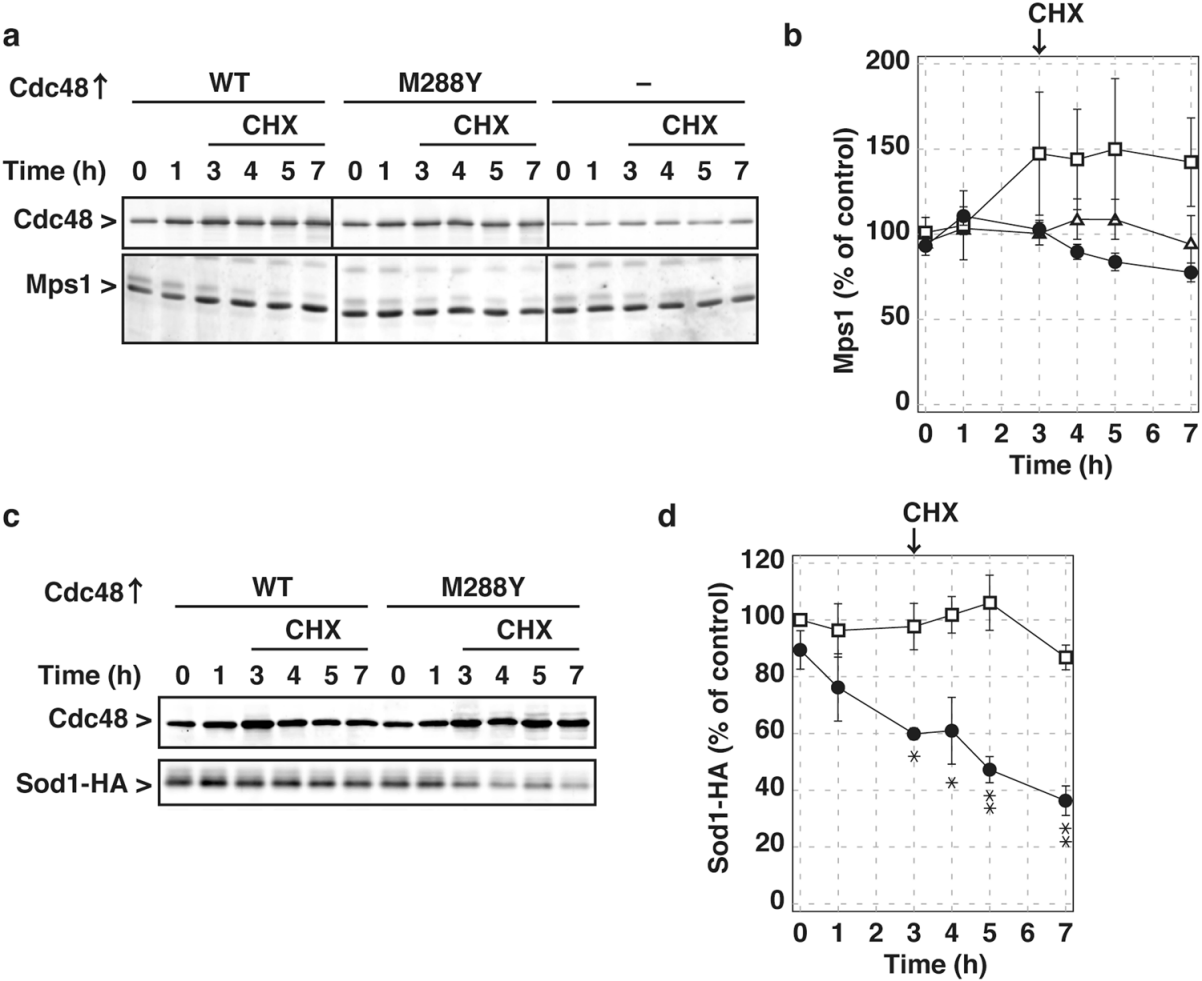
Intracellular proteins were degraded upon expression of the M288Y Cdc48 mutant. (**a**) Wild-type cells were transformed with the *CUP1* promoter-regulated plasmid expressing wild-type Cdc48 (WT), the M288Y Cdc48 mutant, and no proteins. The transformants were grown in a glucose medium at 30 °C. At early log phase, 500 µM CuCl_2_ were added, and the cells were further incubated for 3 h. Then 100 µg/ml cycloheximide (CHX) were added, and incubation was continued for 4 h. Whole cell lysates were prepared at the indicated times as shown previously^48^. Equal amounts of cell extracts were analyzed by SDS-PAGE followed by immunoblotting using anti-Cdc48 and Mps1 antibodies. (**b**) Amounts of Mps1 were quantified. The amounts in the cells without overproduction of Cdc48 were set to 100%. Squares, wild-type Cdc48 overexpressed; circles, the Cdc48 M288Y mutant overexpressed; triangles, no proteins overexpressed. Averages and standard errors from three independent experiments were shown. (**c**) The YME2240 and YME2241 cells were transformed with pME809 expressing Sod1-HA. The transformants were grown in a glucose medium at 30 °C. At early log phase, 200 nM β-estradiol were added, and the cells were further incubated for 3 h. Then 100 µg/ml CHX were added, and incubation was continued for 4 h. Cdc48 and Sod1-HA were analyzed as described above using anti-Cdc48 and HA antibodies. (**d**) Amounts of Sod1-HA were quantified. The amounts in the cells before overproduction of wild-type Cdc48 were set to 100%. Squares, wild-type Cdc48 overexpressed; circles, Cdc48 M288Y mutant overexpressed. Averages and standard errors from three independent experiments were shown. Asterisks **·** and **··** indicate significant differences with P < 0.05 and P < 0.01, respectively, by student t-test.

To directly evaluate whether the Cdc48 M288Φ mutation leads to abnormal degradation of certain proteins, we searched for any such degraded proteins using a cycloheximide-chase analysis. In brief, wild-type and mutant Cdc48 were overproduced by the addition of Cu^2+^ for 3 h, and then cycloheximide was added to inhibit new protein synthesis and protein stability was assessed for an additional 4 h. One of the known substrates of Cdc48, Mps1^34–36^, was found to be affected by expression of the Cdc48 M288Y mutant. In the cycloheximide-chase analysis, Mps1 was relatively stable under normal conditions, but the amount of Mps1 was moderately decreased upon expression of the Cdc48 M288Y mutant (Fig. 5a,b).

We then searched for proteins that would become destabilized in the context of expression of the Cdc48 M288Y mutant using a proteomics approach (unpublished results), and identified Sod1, a cytosolic copper-zinc superoxide dismutase, as a possible candidate. To eliminate the possible effects of Cu^2+^, which was used to induce expression of the Cdc48 M288Y mutant, we constructed another induction system using the LexA-ER-AD system^37^. In brief, a gene encoding a fusion protein of the bacterial LexA DNA-binding protein, human estrogen receptor, and a fragment of the herpes simplex virus type I transactivator VP16 was integrated into the yeast genome. We also integrated gene cassettes composed of the wild-type or mutant *CDC48* genes that were placed downstream of a promoter containing eight *lexA* boxes. Upon addition of β-estradiol, Cdc48 was overproduced in addition to the endogenous Cdc48 copy. HA-tagged Sod1 was relatively stable upon overexpression of wild-type Cdc48 (Fig. 5c,d). In contrast, upon overexpression of the Cdc48 M288Y mutant, HA-tagged Sod1 was significantly degraded (Fig. 5c,d). These results support the idea that the Cdc48 M288Φ mutant accelerates the degradation of certain proteins abnormally.

## Discussion

According to a phylogenetic analysis, AAA proteins can be classified into six major clades^38^. The ΦXG motif sequence is conserved in the pore loop of AAA modules in all clades except for that comprising Bcs1 and its homologs, and is essential for their proper functions. Like other AAA modules, the C-terminal D2 AAA module of Cdc48 possesses a ΦXG motif sequence, which is essential for Cdc48 functions. This finding is consistent with our previous observation that the D2 module is the major functional unit of Cdc48^16^. By contrast, the N-terminal D1 AAA modules of eukaryotic Cdc48 homologs uniquely lack the motif sequence in the pore loop. In this study, we evaluated the essential features of the D1 pore loop of yeast Cdc48, and showed that the addition of artificial ΦXG motif sequences in the D1 pore loop led to cell death. An additional mutation such as an ATPase-deficient mutation or deletion of the C-terminal residues could suppress this lethal phenotype. These findings suggest that occupancy of a non-aromatic amino acid at the first residue of the D1 pore loop is not the essential feature required for maintaining Cdc48 functions; rather, it is likely that possession of an aromatic amino acid at this residue vests a fatal power to Cdc48.

We and other groups have previously shown that Cdc48 hydrolyzes ATP in a positively cooperative manner, and that the loss of this positive cooperativity leads to cell death^17, 39, 40^. Locking the D2 AAA domain in a protomer within a Cdc48 hexamer in an ATP-bound form strongly reduced the whole ATPase activity^17^, which can explain the dominant negative effects of the overproduction of the D2 ATPase activity-deficient Cdc48 mutants in the present study. In particular, the Cdc48 M288Y-expressing cells showed a more severe growth defect than the ATPase-deficient cells. Thus, although the M288Y mutation likely enhanced the ATPase activity of Cdc48, this enhanced activity is not the reason for the lethality given that concomitant deletion of the C-terminal residues also showed enhanced ATPase activity but suppressed the lethality. The severe growth defect observed also supports the idea that M288Φ mutations vest an additional power to Cdc48 rather than perturbing any essential behaviors of the enzyme.

Although removal of the N-terminal regulatory domain is a prerequisite, the *in vitro* unfolding activity was found to be maintained by p97 harboring an artificial ΦXG pore loop in the D1 domain^18^. In addition, the p97∆N mutant harboring the D1 ΦXG pore loop in complex with the 20S proteasome facilitated the degradation of model proteins *in vitro* ^19^. In a similar vein, the lethal phenotype of the Cdc48 mutant with the D1 ΦXG pore loop could be suppressed by deletion of the C-terminal residues and by the addition of the bulky GFP at the C-terminus, both of which are expected to inhibit the interaction of Cdc48 with the 20S proteasome but not the extrinsic unfolding activity of the mutant D1 domain. A mutation resulting in reduced protease activity in a 20S proteasomal subunit also suppressed the lethal phenotype of the Cdc48 M288Y mutant, and intracellular proteins were destabilized upon expression of the Cdc48 M288Y mutant. Taking these results together, we propose that Cdc48 with the D1 ΦXG pore loop may facilitate protein unfolding through the AAA pores and their degradation in conjunction with the 20S proteasome *in vivo*, even when the N-terminal domain is present (Supplementary Fig. S7). The *Thermoplasma* homolog of Cdc48 VAT, which naturally has the ΦXG pore loop in the D1 domain, exhibits unfolding activity in the presence of the N-terminal domain, and removal of the N-terminal domain greatly enhanced its unfolding activity^41^. Therefore, it is reasonable to assume that eukaryotic Cdc48 with the M288Φ mutation possesses threading activity even in the presence of the N-terminal domain.

We also found that Ufd2 is likely involved in the Cdc48 M288Φ lethal phenotype. However, a Cdc48 interaction-defective mutation in Ufd2 did not rescue the Cdc48 M288Φ lethal phenotype. *In vitro* analyses showed that yeast Ufd2 interacts with the C-terminal region of yeast Cdc48, whereas a human homolog of Ufd2, E4B, interacts with the N-terminal domain of p97^31^. If yeast Ufd2 is also able to interact with the N-terminal domain of Cdc48 *in vivo*, this could induce a conformational change of Cdc48 to reduce the steric hindrance of the N-terminal domain for the threading mechanism. In addition to Ufd2, other cofactors such as Shp1 and Ubx4 may also contribute to reducing the steric hindrance, since they preferentially interact with the N-terminal domain of Cdc48. In contrast to the Cdc48 M288Φ mutants, wild-type Cdc48 did not facilitate protein degradation, suggesting that the protein threading may less effectively occur through the wild-type D1 pore. Thus, it is likely that Cdc48 predominantly functions via a new and as-yet-unknown mechanism. Even though less effective, the widely applicable threading mechanism may also be functional for Cdc48 in some circumstances, e.g. selective unfolding of loosely folded substrate proteins. Consistently, a recent report showed that purified wild-type Cdc48 in conjunction with cofactor proteins could unfold polyubiquitinated substrate proteins by the threading mechanism *in vitro*, although the efficiency has not yet been evaluated^42^.

Actual *in vitro* complex formations of purified eukaryotic Cdc48 and the 20S proteasome are not readily detectable without a stabilization step such as chemical cross-linking^22^, although their functional interactions have been observed with micromolar or lower affinity^19, 43^. We have also found that the interaction of Cdc48 and the 20S proteasome occur only transiently using high-speed atomic force microscopy (unpublished observation). Since Ufd2 binds to the C-terminal portion of Cdc48^31^ and was shown to be involved in the Cdc48 M288Φ lethal phenotype, it is possible that Ufd2 may facilitate the formation of the complex of Cdc48 with the 20S proteasome, even though a Ufd2 mutant that was defective in efficient *in vitro* interactions with Cdc48 was also functional in this pathway. The Cdc48–20S proteasome complex does not appear to play any essential roles for vegetative growth since inhibition of the interaction had no impact on cell growth. Thus, the complex may function as a backup system under specific conditions such as under stress, since it can bypass the normal complicated protein degradation pathway, in which substrate proteins should be ubiquitinated, recognized by Cdc48, processed by Ufd2 and Rad23/Dsk2, and finally degraded by the 26S proteasome. It should be noted that the canonical degradation pathway appears to be primarily responsible for abnormal conditions because strains with deletions in the required factors such as Rad23/Dsk2 were shown to be more sensitive to several stresses^44^. Another possible function of the Cdc48–20S proteasome complex is that Cdc48 may mask the 20S proteasome by competitive binding with the 19S regulatory particle to reduce the concentration of the functional 26S proteasome for certain reasons.

The present study provides preliminary indirect evidence for the *in vivo* existence of the Cdc48–20S proteasome complex; further analyses directly showing physical interactions among them and their substrates will be needed to more precisely confirm the existence of the complex. The identified endogenous proteins degraded by the Cdc48 M288Y mutant may be ideal substrates to reveal the biological significance of the new proteasome complex in future analyses.

## Methods

### Strains

All strains used in this study are listed in Supplementary Table S1.

A DNA fragment containing the open reading frame of the *CDC48* gene with 300-bp 5′ and 3′ untranslated regions (UTR) of the budding yeast *Saccharomyces cerevisiae* was inserted into the plasmids pRS316 (*URA3*, *CEN/ARS*) and pRS313 (*HIS3*, *CEN/ARS*)^45^ to generate pME351 and pME368, respectively. Site-specific mutations were introduced into pME368 for expression of Cdc48 mutants. A W303-based haploid strain, YME0115^16^, in which the chromosomal *CDC48* gene was disrupted by the KanMX6 module but wild-type Cdc48 was expressed from pME351^16^, was used for the plasmid shuffling assay. Briefly, YME0115 was transformed with pME368 and its derivatives, and the transformants were selected on SD media (0.67% yeast nitrogen base without amino acids, 2% glucose, appropriate supplements)^46^ without histidine at 30 °C. Then the transformants were streaked on SD media without histidine supplemented with 1 mg/ml 5-fluoroorotic acid to test growth ability of the cells without pME351.

For expression of Cdc48 under the control of the *CUP1* promoter, a DNA fragment containing 500-bp 5′ UTR of the *CUP1* gene was amplified using oligonucleotides 5′–GGTGCTCGAGCTTCAACGATTTCTATGATG–3′ and 5′–ATTTTATGTGATGATTGATTGATTG–3′, and digested with XhoI. The *CDC48* coding region with 300-bp 3′ UTR was amplified using oligonucleotides 5′–CCAAGTCGCCCGGGTGAAGAACATAAACC–3′ and 5′–CAGGAGCTCTAAATGTCGAAATTATGCC–3′, and digested with SmaI and SacI. The two fragments were ligated, and inserted into the XhoI- and SacI-digested pRS313 to generate pME743. Site-specific mutations were introduced into pME743 for expression of Cdc48 mutants. A wild-type haploid strain W303-1 was transformed with pME743 and its derivatives, and the transformants were selected on modified SD media (0.69% yeast nitrogen base without amino acids and without copper (Formedium), 2% glucose, appropriate supplements) without histidine at 30 °C. Addition of Cu^2+^ induced expression of Cdc48.

Cdc48 cofactor-deleted strains were generated from the haploid strain W303-1 to replace the whole coding region of each gene locus with the *LEU2* or *TRP1* gene of *Candida glabrata* (provided by the National Bio-Resource Project, Japan). The *ufd1-1* strain was constructed as follow. A DNA fragment containing the open reading frame of the *UFD1* gene with 300-bp 5′ and 3′ UTRs was inserted into the plasmids pRS316 and pRS314 (*TRP1*, *CEN/ARS*) to generate pME760 and pME761, respectively. Site-specific mutagenesis was performed to replace the codon for Val 94 with one for Asp in pME761, generating pME763. One of the *UFD1* allele of the diploid strain W303 was disrupted by replacement with the KanMX6 gene^47^. The resulting strain was transformed with pME760, and sporulation and dissection of the diploid strain gave a haploid strain YME2120, which expresses Ufd1 only from pME760. YME2120 was transformed with pME761 and pME763, and then pME760 was shuffled out by counterselection on 5-fluoroorotic acid-containing media, to yield YME2126 and YME2127, respectively. The Ufd2 G274D mutant-expressing cells and its control strains were generated as follows. A DNA fragment containing the coding region of the *UFD2* gene and 500-bp 5′ UTR was amplified using oligonucleotides 5′–CTTGCTCGAGGCACGTTTCAACATACTGTC–3′ and 5′–CCCGCTAGCCTCGCTTGCTTTATGTTTTGC–3′, and digested with XhoI and NheI. Another DNA fragment containing a sequence coding the myc tag followed by the *CYC1* terminator was amplified using oligonucleotides 5′–CTGTGCTAGCGGAGAACAAAAGCTGATCAGCGAAGAAGATCTGTAATCATGTAATTAGTTATGTC–3′ and 5′–GTATCCGCGGGCCGCAAATTAAAGCCTTCG–3′, and digested with NheI and SacII. The two digested fragments were ligated into XhoI- and SacII-treated pRS316, yielding pME773. A plasmid pME774 is essentially the same with pME773 but with a mutation in the codon for Gly 274 with one for Asp. The YME2070 (*ufd2∆*) strain was transformed with pME773, pME774, and pRS316 to generate strain expressing myc-tagged wild-type Ufd2 (YME2146), myc-tagged Ufd2 G274D mutant (YME2147), and no Ufd2 proteins (YME2148), respectively.

The *pre4-1* strain was generated from the haploid strain W303-1 as follow. The *CYC1* terminator followed by the *LEU2* gene of *C. glabrata* was cloned into the XmaI and XhoI-digested pBluescript II plasmid, yielding pME644. The coding region of the *pre4-1* allele, which contains a nonsense mutation in the codon 252, with the 290-bp 5′ UTR was inserted in front of the *CYC1* terminator of pME644, yielding pME804. A DNA cassette containing the *pre4-1* gene followed by the *CgLEU2* gene was amplified from pME804 using oligonucleotides 5′–CCCGGGATCCTACATTCACATATAAAATAC–3′ and 5′–GATGATATTATGAATTGAAAAATAAAAATAAAATGAGTATTTAATGAAGGTGGGATAAAACTCGTAAAACGACGGCCAGT–3′, and used to transform the haploid strain W303-1. Transformants containing a wild-type *PRE4* and mutant *pre4-1* alleles were selected, and named YME2192 and YME2193, respectively.

A plasmid expressing Sod1-HA, pME809, was constructed as follow. A DNA fragment containing the coding region of the *UFD2* gene and 300-bp 5′ UTR was amplified using oligonucleotides 5′–GTGTCCCGGGGCGTGCGACTCACCCACTCA–3′ and 5′–CTCTCTAGCGTTGGTTAGACCAATGACACC–3′, and digested with XmaI and NheI. Another DNA fragment containing a sequence coding the two HA tag followed by the *CYC1* terminator was amplified using oligonucleotides 5′–GCTAGCTACCCATATGACGTTCCAGACTACGCGTACCCTTACGACGTACCTGACTACGCTTAATCATGTAATTAGTTATG–3′ and 5′–CAGAGAGCTCCGCAAATTAAAGCCTTCGAG–3′, and digested with NheI and SacI. The two digested fragments were ligated into XmaI- and SacI-treated pRS315.

Strains expressing Cdc48 using the LexA-ER-AD system^37^ were constructed as follow. The promoter region containing eight *lexA* boxes was retrieved by digesting FRP795 with NotI and XmaI. The *CDC48* coding region and the 3′ UTR was amplified using oligonucleotides 5′–ATGCCCGGGGCAGCATGGGTGAAGAACATA–3′ and 5′–CAGGAGCTCTAAATGTCGAAATTATGCC–3′, and digested with XmaI and SacI. The two fragments were ligated and inserted into XmaI- and SacI-treated pRS306, yielding pME829. A site-specific mutation was introduced into pME829 for expression of the Cdc48 M288Y mutant, yielding pME830. The W303a strain was transformed with SbfI-linearized pME829 and pME830 together with NheI-linearized FRP467 (Addgene) to integrate the transcription factor LexA-ER-VP60^37^ into the *HIS3* locus, yielding YME2240 and YME2241, respectively.

### Pull down assay

DNA fragments coding wild-type Cdc48 and the Cdc48 M288Y mutant with and without deletion of the C-terminally 1 and 2 amino acid residues, ∆S and ∆YS, respectively, were cloned in pET15b. N-terminally His-tagged Cdc48 were expressed in BL21(DE3), and purified by HisTrap affinity chromatography followed by DEAE column chromatography. Purified Cdc48 were stored in 20 mM Tris-HCl, pH 7.4, 50 mM NaCl, 1 mM MgCl_2_, 5 mM β-mercaptoethanol, and 20% glycerol. A DNA fragment coding Ufd2 was cloned in pGEX6P-1. GST and GST-Ufd2 were expressed in BL21, and purified using glutathione-sepharose. Purified GST proteins were stored in 20 mM Tris-HCl, pH 7.4, 50 mM NaCl, 5 mM β-mercaptoethanol, and 20% glycerol. GST and GST-Ufd2 (40 pmol) were incubated with 20 pmol Cdc48 in 100 µL of the Cdc48 storage buffer for 10 min at 25 °C, and were further incubated with glutathione-sepharose 4B for 30 min at room temperature. After washing the sepharose beads with the reaction buffer, bound proteins were eluted with 20 mM glutathione, 30 mM Tris-HCl, pH 8.5.

### Data availability

The data that support the findings of the current study are available from the corresponding author on reasonable request.

## Electronic supplementary material

Supplementary information
