# Supplementary material for: Deviation of the typical AAA substrate-threading pore prevents fatal protein degradation in yeast Cdc48
Source: Sci Rep. 2017 Jul 14;7:5475. doi: 10.1038/s41598-017-05806-y (PMC5511170; doi:10.1038/s41598-017-05806-y)
Supplement: Supplementary file 1 — Supplementary information [file 41598_2017_5806_MOESM1_ESM.pdf]

Supplementary information

Deviation of the typical AAA substrate-threading pore prevents fatal protein degradation in yeast Cdc48

Masatoshi Esaki, Md. Tanvir Islam, Naoki Tani, and Teru Ogura

```
                 Walker A                                 pore                          Walker B                                                   SRH
Cdc48_N 250 GVLMYGPPGTGKTLMARAVAN-----ETGAFFFLINGP----EVMSKMAGESESNLRKAFEEAEKNAPA-----IIFIDEIDSIAPKRD----KTN-GEVERRVVSQLLTLMDGMK-------ARSN-VVVIAATNRPNSIDPALRRFGRFDR 375
Cdc48_C 523 GVLFYGPPGTGKTLLAKAVAT-----EVSANFISVKGP----ELLSMWYGESESNIRDIFDKARAAAPT-----VVFLDELDSIAKARGGS--LGDAGGASDRVVNQLLTEMDGMN-------AKKN-VFVIGATNRPDQIDPAILRPGRLDQ 651
Afg2_N  281 GILLHGPPGTGKTMLLRVVAN-----TSNAHVLTINGP----SIVSKYLGETEAALRDIFNEARKYQPS-----IIFIDEIDSIAPNRA----NDDSGEVESRVVATLLTLMDGMG-------AAGK-VVVIAATNRPNSVDPALRRPGRFDQ 407
Afg2_C  552 GVLLYGPPGCSKTLTAKALAT-----ESGINFLAVKGP----EIFNKYVGESERAIREIFRKARSAAPS-----IIFFDEIDALSPDRD-----GSSTSAANHVLTSLLNEIDGVE-------ELKG-VVIVAATNRPDEIDAALLRPGRLDR 677
Afg3    323 GAILSGPPGTGKTLLAKATAG-----EANVPFLSVSGS----EFVEMFVGVGASRVRDLFTQARSMAPS-----IIFIDEIDAIGKERGKGGALGGANDEREATLNQLLVEMDGFT--------TSDQVVVLAGTNRPDVLDNALMRPGRFDR 453
Bcs1    262 GYLLYGPPGSGKTSFIQALAG-----ELDYNICILNLS-------------ENNLTDDRLNHLMNNMPERS---ILLLEDIDAAFNKRSQT---GEQGFHSSVTFSGLLNALDGVT--------SSEETITFMTTNHPEKLDAAIMRPGRIDY 382
Msp1    128 GVLLYGPPGCGKTMLAKALAK-----ESGANFISIRMS----SIMDKWYGESNKIVDAMFSLANKLQPC-----IIFIDEIDSFLRERS-STDHEVTATLKAEFMTLWDGLLN------------NGRVMIIGATNRINDIDDAFLR--RLPK 251
Pch2    309 LLLVHGPPGTGKTTLCKALCQ(12) TIDTNYKGIIIELSCARIFSKWFGESSKNISIVFKDIEELLKV (8) CLLIDEVEAIASSRTNLS-SRNESTDGIRVVNTLLTQLDRLK--------KYHNFLALATSNLLDSLDDAFVD--RADG 461
Pex1    733 GILLYGYPGCGKTLLASAVAQ-----QCGLNFISVKGP----EILNKFIGASEQNIRELFERAQSVKPC-----ILFFDEFDSIAPKRG-----HDSTGVTDRVVNQLLTQMDGAE-------GLDG-VYILAATSRPDLIDSALLRPGRLDK 858
Pex6    761 GILFYGPPGTGKTLMAKAIAT-----NFSLNFFSVKGP----ELLNMYIGESEANVRRVFQKAREAKPC-----VIFFDEIDSVAPKRG-N--QGDSGGVMDRIVSQLLAELDGMS-------TDADGVFVIGATNRPDLLDEALLRPGRFDK 895
Rix7_N  241 GVLLHGPPGCGKTSIANALAG-----ELQVPFISISAP----SVVSGMSGESEKKIRDLFDEARSLAPC-----LVFFDEIDAITPKRDGG----AQREMERRIVAQLLTSMDELTME----KTNGKPVIIIGATNRPDSLDAALRRAGRFDR 371
Rix7_C  569 GVLLWGPPGCGKTLLAKAVAN-----ESRANFISIKGP----ELLNKYVGESERSIRQVFTRARASVPC-----VIFFDELDALVPRRDTS--LSES---SSRVVNTLLTELDGLN-------DRRG-IFVIGATNRPDMIDPAMLRPGRLDK 694
Rpt1    245 GILLYGPPGTGKTLCARAVAN-----RTDATFIRVIGS----ELVQKYVGEGARMVRELFEMARTKKAC-----IIFFDEIDAVGGARFDD--GAGGDNEVQRTMLELITQLDGFD-------PRGN-IKVMFATNRPNTLDPALLRPGRIDR 373
Rpt6    184 GVILYGPPGTGKTLLARAVAH-----HTDCKFIRVSGA----ELVQKYIGEGSRMVRELFVMAREHAPS-----IIFMDEIDSIGSTRVEG--SGGGDSEVQRTMLELLNQLDGFE-------TSKN-IKIIMATNRLDILDPALLRPGRIDR 312
Rpt5    217 GALMYGPPGTGKTLLARACAA-----QTNATFLKLAAP----QLVQMYIGEGAKLVRDAFALAKEKAPT-----IIFIDELDAIGTKRFDS--EKSGDREVQRTMLELLNQLDGFS-------SDDR-VKVLAATNRVDVLDPALLRSGRLDR 345
Rpt2    219 GVILYGAPGTGKTLLAKAVAN-----QTSATFLRIVGS----ELIQKYLGDGPRLCRQIFKVAGENAPS-----IVFIDEIDAIGTKRYDS--NSGGEREIQRTMLELLNQLDGFD-------DRGD-VKVIMATNKIETLDPALIRPGRIDR 346
Rpt3    208 GVLLYGPPGTGKTMLVKAVAN-----STKAAFIRVNGS----EFVHKYLGEGPRMVRDVFRLARENAPS-----IIFIDEVDSIATKRFDA--QTGSDREVQRILIELLTQMDGFD-------QSTN-VKVIMATNRADTLDPALLRPGRLDR 336
Rpt4    217 GVLLYGPPGTGKTLLAKAVAA-----TIGANFIFSPAS----GIVDKYIGESARIIREMFAYAKEHEPC-----IIFMDEVDAIGGRRFSE--GTSADREIQRTLMELLTQMDGFD-------NLGQ-TKIIMATNRPDTLDPALLRPGRLDR 345
Sap1    640 GMLLFGPPGTGKTMLARAVAT-----ESHSTFFSISAS----SLTSKYLGESEKLVRALFAIAKKLSPS-----IIFVDEIDSIMGSRNNENENESSRRIKNEFLVQWSSLSSAAAG (14) EDDTRVLVLAATNLPWSIDEAARR--RFVR 784
Sec18   553 SLLIHGPAGSGKTALAAEIAL-----KSGFPFIRLISPN---ELSGMSESAKIAYIDNTFRDAYKSPLN-----ILVIDSLETLVDWVPIG--PRFS----NNILQMLKVALKRKP-------PQDRRLLIMTTTSAYSVLQQMDILSCFDNE 685
Yme1    316 GVLLTGPPGTGKTLLARATAG-----EAGVDFFFMSGS----EFDEVYVGVGAKRIRDLFAQARSRAPA-----IIFIDELDAIGGKRNPK----DQ-AYAKQTLNQLLVELDGFS------QTSG--IIIIGATNFPEALDKALTRPGRFDK 441
Yta6    506 GMLLFGPPGTGKTMIAKAVAT-----ESNSTFFSVSAS----SLLSKYLGESEKLVRALFYMAKKLSPS-----IIFIDEIDSMLTARS-DNENESSRRIKTELLIQWSSLSSATAQSEDRNNTLDSRVLVLGATNLPWAIDDAARR--RFSR 641
Yta7    442 GVLFHGPPGTGKTLMARALAASCSSDERKITFFMRKGA----DILSKWVGEAERQLRLLFEEAKKHQPS-----IIFFDEIDGLAPVRS----SKQ-EQIHASIVSTLLALMDGMD-------NRGQ-VIVIGATNRPDAVDPALRRPGRFDR 579
Yta12   383 GAILSGPPGTGKTLLAKATAG-----EAGVPFYFVSGS----EFVEMFVGVGAARVRDLFKTARENAPS-----IVFIDEIDAIGKARQKG-NFSGANDERENTLNQMLVEMDGFT--------PADHVVVLAGTNRPDILDKALLRPGRFDR 512
Vps4    168 GILLYGPPGTGKSYLAKAVAT-----EANSTFFSVSSS----DLVSKWMGESEKLVKQLFAMARENKPS-----IIFIDEVDALTGTRG-EGESEASRRIKTELLVQMNGVGNDSQG-----------VLVLGATNIPWQLDSAIRR--RFER 292
              :. * .* .*:      .                                       :               :..:..:                     .       .                          .  :.    ::
```

Supplementary Figure S1. A sequence alignment of AAA modules found in the budding yeast *Saccharomyces cerevisiae*.

```
                                                                            N domain
Sc  MGEEHKPLLDASGVDPREEDKTATAILRRKKKDNMLLVDDAINDDNSVIAINSNTMDKLELFRGDTVLVKGKKRKDTVLIVLIDDELEDGACRINRVVRN 100
Tb  --------------------MSAKAQQVKQQKLNRLIADDLAEDDNSVVVLNAKRMDELSIFRGDTVKLKGKKNRSTICIAMSDENCPEGSIMVNKVTRR 80
Ce  --MASVP--THQSEKEKKNDELSTAILKDKVKPNRLIVDQSEQDDNSVIAVSQAKMDELGLFRGDAVILKGKKRKESVAIIVSDESCPNEKVRMNRVVRN 96
At  ---MSTP--A-ESSDSKSKKDFSTAILERKKSPNRLVVDEAINDDNSVVSLHPATMEKLQLFRGDTILIKGKKRKDTVCIALADETCEEPKIRMNKVVRS 94
Mm  ----------MASGADSKGDDLSTAILKQKNRPNRLIVDEAINEDNSVVSLSQPKMDELQLFRGDTVLLKGKKRREAVCIVLSDDTCSDEKIRMNRVVRN 90
Hs  ----------MASGADSKGDDLSTAILKQKNRPNRLIVDEAINEDNSVVSLSQPKMDELQLFRGDTVLLKGKKRREAVCIVLSDDTCSDEKIRMNRVVRN 90
                                  :.*    :   * *:.*:  ::****: :    *::* :****:: :****.:.:: * : *:   :    :*:*.*

                                                     N domain
Sc  NLRIRLGDLVTIHPCPDIKYATRISVLPIADTIEGITGNLFDVFLKPYFVEAYRPVRKGDHFVVRGGMRQVEFKVVDVEPEEYAVVAQDTIIHWEGEPIN 200
Tb  NIRILLGDLITVSSHSNVPYGNHVHVLPIDDTVKNLTGDLFETFLKPYFLEAYRPVKTGDLFICRGAMRSVEFKVVEVDPGDCCIVAPETVVHCEGDPIR 180
Ce  NLRIRLGDVVSITPAPNLSYGTRIHVLPIDDTIEGLTGNLFDVFLKPYFLEAYRPLHKGDIFTVQAAMRTVEFKVVETEPAPACIVSPDTMIHYEGDPIK 196
At  NLRVRLGDVISVHQCPDVKYGKRVHILPVDDTVEGVTGNLFDAYLKPYFLEAYRPVRKGDLFLVRGGMRSVEFKVIETDPAEYCVVAPDTEIFCEGEPVK 194
Mm  NLRVRLGDVISIQPCPDVKYGKRIHVLPIDDTVEGITGNLFEVYLKPYFLEAYRPIRKGDIFLVRGGMRAVEFKVVETDPSPYCIVAPDTVIHCEGEPIK 190
Hs  NLRVRLGDVISIQPCPDVKYGKRIHVLPIDDTVEGITGNLFEVYLKPYFLEAYRPIRKGDIFLVRGGMRAVEFKVVETDPSPYCIVAPDTVIHCEGEPIK 190
    *:*: ***::::    :: *..:: :**: **:: :**:**:.:*****:*****::.** *  :..** *****::.:*   .:*: :* :. **:*:.

    N domain                                         D1 AAA domain                           pore
Sc  REDEENNMNEVGYDDIGGCRKQMAQIREMVELPLRHPQLFKAIGIKPPRGVLMYGPPGTGKTLMARAVANETGAFFFLINGPEVMSKMAGESESNLRKAF 300
Tb  REDE-ERLDDVGYDDIGGCRRQLVQIREMVELPIRHPELFKSIGIKPPRGILMYGPPGSGKTLIARAVANETGAFFFLINGPEIMSKMAGESEGNLRNAF 279
Ce  REEEEESMNDIGYDDLGGVRKQLAQIKEMVELPLRHPQLFKAIGIKPPRGILLFGPPGTGKTLIARAVANETGSFFFLINGPEVMSKMSGESESNLRKAF 296
At  REDE-ERLDDVGYDDVGGVRKQMAQIRELVELPLRHPQLFKSIGVKPPKGILLYGPPGSGKTLIARAVANETGAFFFCINGPEIMSKLAGESESNLRKAF 293
Mm  REDEEESLNEVGYDDIGGCRKQLAQIKEMVELPLRHPALFKAIGVKPPRGILLYGPPGTGKTLIARAVANETGAFFFLINGPEIMSKLAGESESNLRKAF 290
Hs  REDEEESLNEVGYDDIGGCRKQLAQIKEMVELPLRHPALFKAIGVKPPRGILLYGPPGTGKTLIARAVANETGAFFFLINGPEIMSKLAGESESNLRKAF 290
    **:* : ::::****:** *:*:.**:*:****:*** ***:**:***:*:*::****:****:*********:*** *****:***::****.***:**

                                                 D1 AAA domain
Sc  EEAEKNAPAIIFIDEIDSIAPKRDKTNGEVERRVVSQLLTLMDGMKARSNVVVIAATNRPNSIDPALRRFGRFDREVDIGIPDATGRLEVLRIHTKNMKL 400
Tb  VESEKNAPAIIFIDEIDSIAPKREKAQGEVEKRIVSQLLTLMDGLKGRSQVIVMAATNRPNAIDPALRRFGRFDREIDIGVPDEIGRLEILRIHTKNMKL 379
Ce  EECEKNQPAILFIDEIDAIAPKREKTNGEVERRIVSQLLTLMDGVKGRSNLVVIAATNRPNSIDGALRRFGRFDREIDIGIPDAVGRLEILRIHTKNMKL 396
At  EEAEKNAPSIIFIDEIDSIAPKREKTNGEVERRIVSQLLTLMDGLKSRAHVIVMGATNRPNSIDPALRRFGRFDREIDIGVPDEIGRLEVLRIHTKNMKL 393
Mm  EEAEKNAPAIIFIDELDAIAPKREKTHGEVERRIVSQLLTLMDGLKQRAHVIVMAATNRPNSIDPALRRFGRFDREVDIGIPDATGRLEILQIHTKNMKL 390
Hs  EEAEKNAPAIIFIDELDAIAPKREKTHGEVERRIVSQLLTLMDGLKQRAHVIVMAATNRPNSIDPALRRFGRFDREVDIGIPDATGRLEILQIHTKNMKL 390
    *.*** *:*:****:*:*****:*:.****:*:**********:* *:.::*:.******:** ***********:***:**  ****:*:********

                                        D1 AAA domain                           D2 AAA domain
Sc  ADDVDLEALAAETHGYVGADIASLCSEAAMQQIREKMDLIDLDEDEIDAEVLDSLGVTMDNFRFALGNSNPSALRETVVESVNVTWDDVGGLDEIKEELK 500
Tb  DPNVDVEKIAKDSHGYVGADLAQLCTEAAMQCVREKMAVVDWDDETIDAEVLDSMSVTNNHFLDALSKMNPSALRETQVETPNVTWSDVGGLLDVKRELQ 479
Ce  ADDVDLEQIANECHGFVGADLASLCSEAALQQIREKMELIDLEDDQIDAEVLNSLAVTMENFRFAQGKSSPSALREAVVETPNTTWSDIGGLQNVKRELQ 496
At  AEDVDLERISKDTHGYVGADLAALCTEAALQCIREKMDVIDLEDDSIDAEILNSMAVTNEHFHTALGNSNPSALRETVVEVPNVSWNDIGGLENVKRELQ 493
Mm  ADDVDLEQVANETHGHVGADLAALCSEAALQAIRKKMDLIDLEDETIDAEVMNSLAVTMDDFRWALSQSNPSALRETVVEVPQVTWEDIGGLEDVKRELQ 490
Hs  ADDVDLEQVANETHGHVGADLAALCSEAALQAIRKKMDLIDLEDETIDAEVMNSLAVTMDDFRWALSQSNPSALRETVVEVPQVTWEDIGGLEDVKRELQ 490
       :**:* :: : **.****:* **:***:* :*:** ::* :::  ****:::*:.** :.*  * .: .******: **  :.:*.*:*** ::*.**:

                                                 D2 AAA domain  pore
Sc  ETVEYPVLHPDQYTKFGLSPSKGVLFYGPPGTGKTLLAKAVATEVSANFISVKGPELLSMWYGESESNIRDIFDKARAAAPTVVFLDELDSIAKARGGSL 600
Tb  ELVQYPVEFPWKFEKYGISAPKGVLFYGPPGCGKTLLAKAIATECQANFISIKGPELLTMWFGESEANVRDVFDKARAAAPCVLFFDELDSVARSRGHS- 578
Ce  ELVQYPVEHPEKYLKFGMQPSRGVLFYGPPGCGKTLLAKAIANECQANFISIKGPELLTMWFGESEANVRDVFDKARAAAPCVLFFDELDSIAKARGGGA 596
At  ETVQYPVEHPEKFEKFGMSPSKGVLFYGPPGCGKTLLAKAIANECQANFISVKGPELLTMWFGESEANVREIFDKARQSAPCVLFFDELDSIATQRGGGS 593
Mm  ELVQYPVEHPDKFLKFGMTPSKGVLFYGPPGCGKTLLAKAIANECQANFISIKGPELLTMWFGESEANVREIFDKARQAAPCVLFFDELDSIAKARGGNI 590
Hs  ELVQYPVEHPDKFLKFGMTPSKGVLFYGPPGCGKTLLAKAIANECQANFISIKGPELLTMWFGESEANVREIFDKARQAAPCVLFFDELDSIAKARGGNI 590
    * *:***  .* :: *:*:    :********* ********:*.* .*****:******:**:****:*:*::*****  :** *:*:*****:*  **

                                                 D2 AAA domain
Sc  -GDAGGASDRVVNQLLTEMDGMNAKKNVFVIGATNRPDQIDPAILRPGRLDQLIYVPLPDENARLSILNAQLRKTPLEPGLELTAIAKATQGFSGADLLY 699
Tb  --GDGGASDRVINQILTEMDGMNSKKNVFIIGATNRPDVLDPAVMRPGRLDQLIYIPLPDKASRVAILKASFRKSPLAPDVDLDQLAAATHGFSGADLAG 676
Ce  GGDGGGASDRVINQVLTEMDGMNAKKNVFIIGATNRPDIIDPAVLRPGRLDQLIYIPLPDEASRHQILKASLRKTPLSKDLDLTFLAKNTVGFSGADLTE 696
At  GGDGGGAADRVLNQLLTEMDGMNAKKTVFIIGATNRPDIIDSALLRPGRLDQLIYIPLPDEDSRLNIFKAALRKSPIAKDVDIGALAKYTQGFSGADITE 693
Mm  -GDGGGAADRVINQILTEMDGMSTKKNVFIIGATNRPDIIDPAILRPGRLDQLIYIPLPDEKSRVAILKANLRKSPVAKDVDLEFLAKMTNGFSGADLTE 689
Hs  -GDGGGAADRVINQILTEMDGMSTKKNVFIIGATNRPDIIDPAILRPGRLDQLIYIPLPDEKSRVAILKANLRKSPVAKDVDLEFLAKMTNGFSGADLTE 689
          ***:***:**:*******.:**.**:********* :* *::**********:****: :*  *::* :**:*:   :::  :*  * ******:

                                    D2 AAA domain
Sc  IVQRAAKYAIKDSIEAHRQHEAEKEVKVEGEDVEMTDEGAKAEQEPEVDPVPYITKEHFAEAMKTAKRSVSDAELRRYEAYSQQMKASRGQFSNFNFNDA 799
Tb  ICQRACKLAIRESIAKEIQLEEAR--------ANG-----VLNEDQDIDPVPQITRLHVEEAMRGARRSVSDADIRKYELFATSLQQSRAFGNVSLDPGN 763
Ce  ICQRACKLAIRESIEKEIRIEKER--------QDRQARGEELMEDDAVDPVPEITRAHFEEAMKFARRSVTDNDIRKYEMFAQTLQQSRGFGNNFKFPGE 788
At  ICQRACKYAIRENIEKDIEKEKRR--------SENP----EAMEEDGVDEVSEIKAAHFEESMKYARRSVSDADIRKYQAFAQTLQQSRGFGSEFRFENS 781
Mm  ICQRACKLAIRESIESEIRRERER--------QTNP----SAMEVEEDDPVPEIRRDHFEEAMRFARRSVSDNDIRKYEMFAQTLQQSRGFGS-FRFPSG 776
Hs  ICQRACKLAIRESIESEIRRERER--------QTNP----SAMEVEEDDPVPEIRRDHFEEAMRFARRSVSDNDIRKYEMFAQTLQQSRGFGS-FRFPSG 776
    * ***.* **::.*  . . *  :                    :    * *  *   *. *:*: *:***:* ::*:*: ::  :: **.  .

                                      HbYX
Sc  PLGTTATDNANSNNSAPSGAGAAFG---SNAEEDDDLYS  835     Sc: Saccharomyces cerevisiae
Tb  QG-GAGGDA---------G-----------AGDDDLYS  780     Tb: Trypanosoma brucei
Ce  QRGSDAPSA---------PVPA---------QDDDDLYN  809     Ce: Caenorhabditis elegans
At  AGSGAT--T---------GVADPFATSAAAAGDDDDLYN  809     At: Arabidopsis thaliana
Mm  NQGGAGPSQ---------GSGGGTGGSVYTEDNDDDLYG  806     Mm: Mus musculus
Hs  NQGGAGPSQ---------GSGGGTGGSVYTEDNDDDLYG  806     Hs: Hopo sapience
                                     *****
```

Supplementary Figure S2. A sequence alignment of eukaryotic Cdc48 homologs.

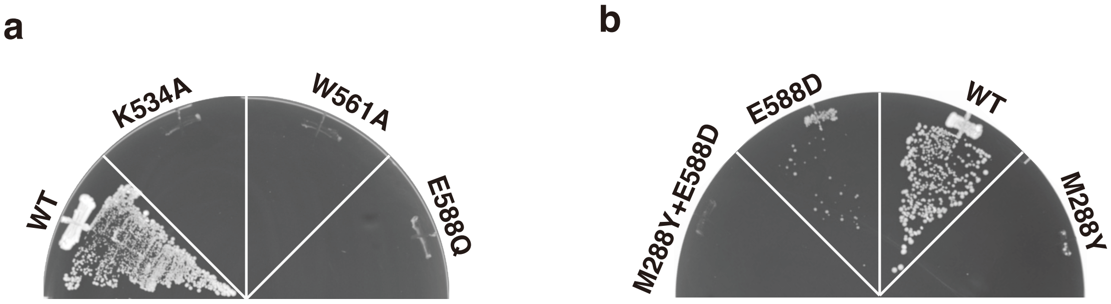

Supplementary Figure S3. Tested were viabilities of cells expressing Cdc48 with the indicated mutations as shown in Fig. 1b.

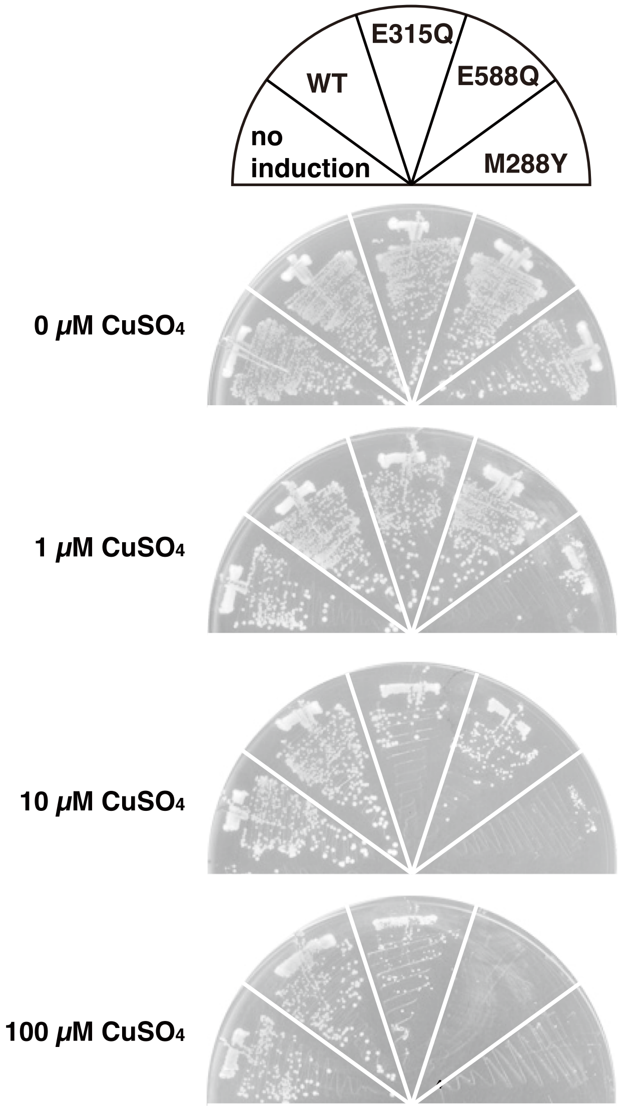

Supplementary Figure S4. Wild type cells were transformed with a *CUP1* promoter-regulated plasmid expressing wild type Cdc48 (WT), mutant Cdc48 with the replacement of E315Q, E588Q, and M288Y, and no proteins. The transformants were grown on media supplemented with $CuSO_4$ at 30°C for 2 days.

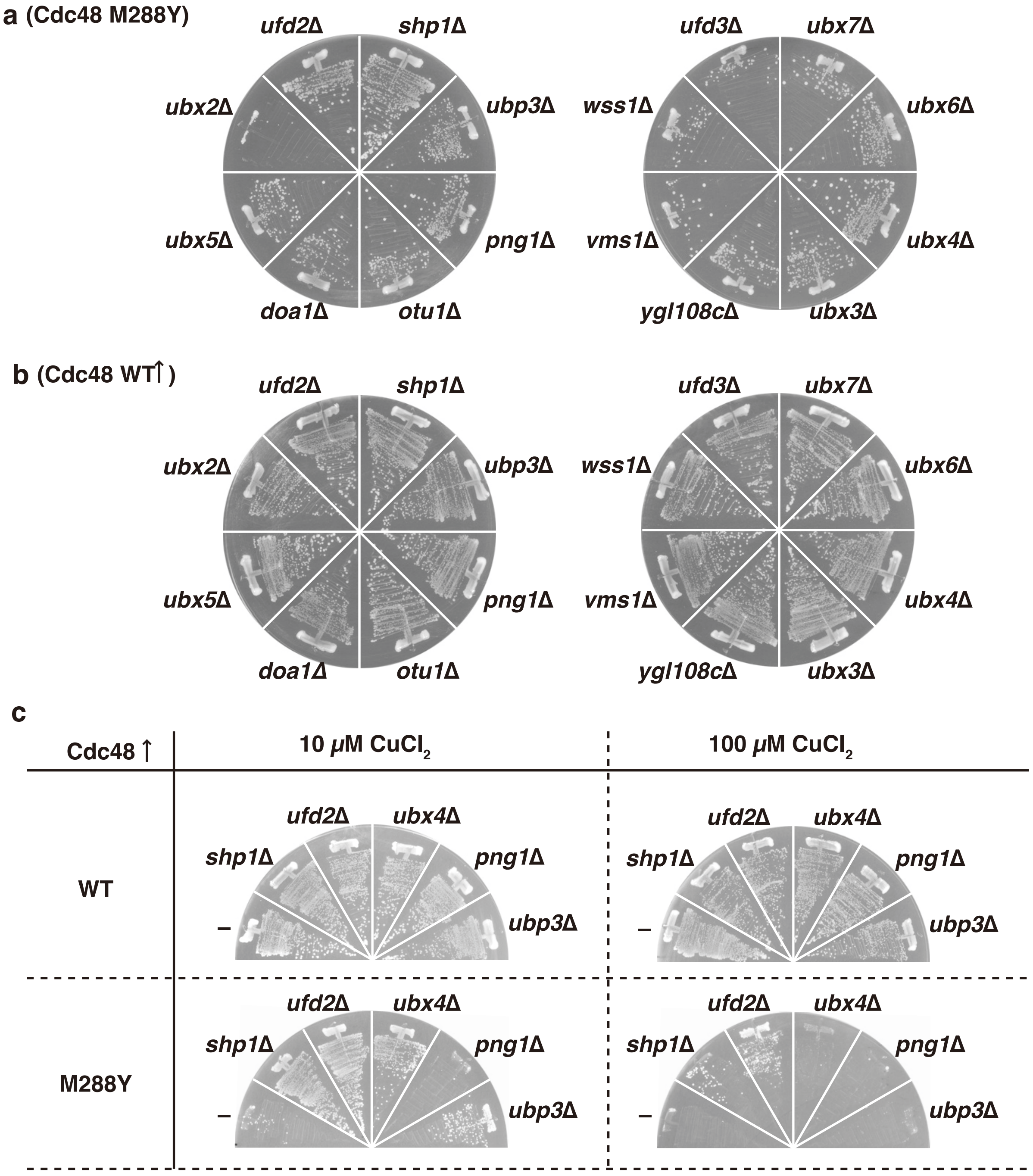

Supplementary Figure S5. a, cells lacking the indicated endogenous gene were transformed with a plasmid expressing the Cdc48 M288Y mutant under the control of the *CUP1* promoter. The transformants were grown on media in the absence $CuCl_2$ at 30°C for 2 days. b, cells lacking the indicated endogenous gene were transformed with a plasmid expressing the wild type Cdc48 under the control of the *CUP1* promoter. The transformants were grown on media supplemented with 10 μM $CuCl_2$ at 30°C for 2 days. c, cells lacking the indicated endogenous gene were transformed with a *CUP1* promoter-regulated plasmid expressing wild type Cdc48 (WT) and mutant Cdc48 with the replacement of M288Y. The transformants were grown on media supplemented with $CuCl_2$ at 30°C for 2 days.

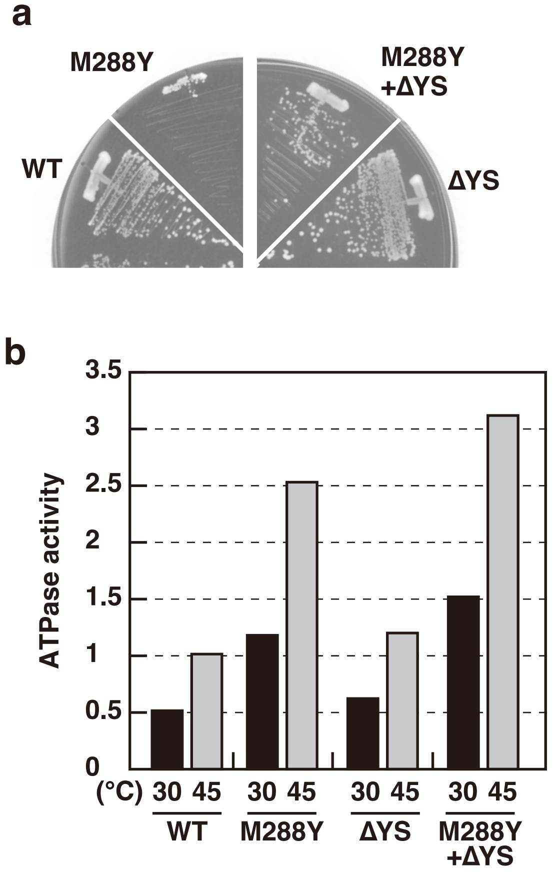

Supplementary Figure S6. a, tested were viabilities of cells expressing Cdc48 with the indicated mutations as shown in Fig. 1b. ΔYS, deletion of the C-terminal 2 amino acid residues of Cdc48. b, ATPase activity of Cdc48 was increased by the M288Y mutation. Recombinant Cdc48 of wild type (WT) and with the M288Y mutation and deletion of the C-terminal two amino acid residues (ΔYS) and both (M288Y+ΔYS). The ATPase activity (μmol/min/mg) was measured at 30°C and 45°C by the malachite green assay described previously (Nishikori et al., 2011). c, peptidase activity of the 20S proteasome was enhanced by Cdc48 in a C-terminal two amino acid residues-dependent manner. A synthetic model peptide, Mca-RPPGFSAFK(Dnp)-OH (R&D Systems), was used to measure the peptidase activity of the yeast 20S proteasome in the absence of nucleotides as described previously (Barthelme and Sauer, 2013). Enhancment factors of the peptidase activity by ~20-fold higher concentration of yeast Cdc48 were shown.

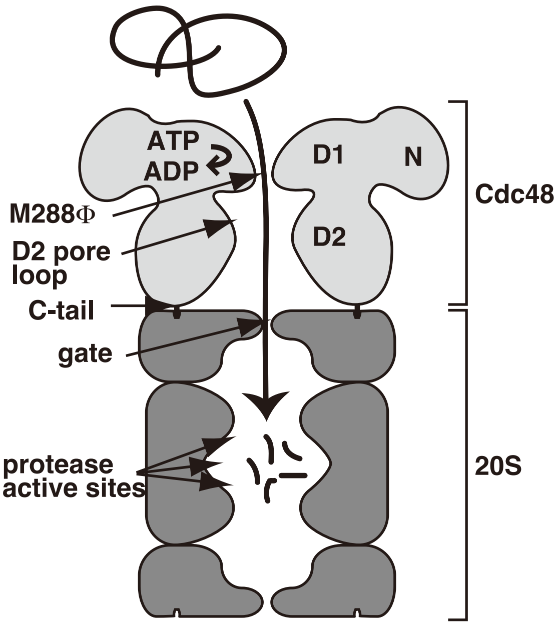

Supplementary Figure S7. Proposed model of protein degradation by the Cdc48-20S proteasome complex.

Supprementary Table S1. Yeast strains used in this study

Strain	Genotype	References
W303	*leu2-3,112,/leu2-3,112, trp1-1/trp1-1, can1-100/can1-100, ura3-1/ura3-1, ade2-1/ade2-1, his3-11,15/his3-11,15*	Lab stock
W303-1	*leu2-3,112, trp1-1, can1-100, ura3-1, ade2-1, his3-11,15*	Lab stock
YME0115	W303-1, *cdc48Δ::KanMX6*, [pME351 (*CDC48*, *URA3*)]	Esaki and Ogura (2010)
YME2063	W303-1, *shp1Δ::CgTRP1*	This study
YME2064	W303-1, *ubx2Δ::CgTRP1*	This study
YME2065	W303-1, *ubx5Δ::CgTRP1*	This study
YME2066	W303-1, *doa1Δ::CgTRP1*	This study
YME2067	W303-1, *otu1Δ::CgTRP1*	This study
YME2068	W303-1, *png1Δ::CgTRP1*	This study
YME2069	W303-1, *ubp3Δ::CgTRP1*	This study
YME2070	W303-1, *ufd2Δ::CgTRP1*	This study
YME2071	W303-1, *ufd3Δ::CgTRP1*	This study
YME2072	W303-1, *wss1Δ::CgTRP1*	This study
YME2106	W303-1, *vms1Δ::CgTRP1*	This study
YME2108	W303-1, *ygl108Δ::CgTRP1*	This study
YME2110	W303-1, *ubx3Δ::CgTRP1*	This study
YME2112	W303-1, *ubx4Δ::CgTRP1*	This study
YME2114	W303-1, *ubx6Δ::CgTRP1*	This study
YME2116	W303-1, *ubx7Δ::CgTRP1*	This study
YME2120	W303-1, *ufd1Δ::KanMX6*, [pME760 (*UFD1*, *URA3*)]	This study
YME2126	W303-1, *ufd1Δ::KanMX6*, [pME761 (*UFD1*, *TRP1*)]	This study
YME2127	W303-1, *ufd1Δ::KanMX6*, [pME763 (*ufd1-1*, *TRP1*)]	This study
YME2146	W303-1, *ufd2Δ::CgTRP1*, [pME773 (*UFD2-MYC*, *URA3*)]	This study
YME2147	W303-1, *ufd2Δ::CgTRP1*, [pME774 (*ufd2-G274D-myc*, *URA3*)]	This study
YME2148	W303-1, *ufd2Δ::CgTRP1*, [pRS316 (*URA3*)]	This study
YME2149	W303-1, *rad23Δ::CgLEU2*	This study
YME2151	W303-1, *dsk2Δ::CgTRP1*	This study
YME2155	W303-1, *rad23Δ::CgLEU2*, *dsk2Δ::CgTRP1*	This study
YME2192	W303-1, *PRE4::CgLEU2*	This study
YME2193	W303-1, *pre4-1::CgLEU2*	This study
YME2240	W303-1, *HIS3::LexA-ER-VP60*, *URA3::*P_{LEXA8}*-CDC48 WT*	This study
YME2241	W303-1, *HIS3::LexA-ER-VP60, URA3::*P_{LEXA8}*-CDC48 M288Y*	This study
